# Fast imaging of live organisms with sculpted light sheets

**DOI:** 10.1038/srep09385

**Published:** 2015-04-20

**Authors:** Aleksander K. Chmielewski, Anders Kyrsting, Pierre Mahou, Matthew T. Wayland, Leila Muresan, Jan Felix Evers, Clemens F. Kaminski

**Affiliations:** 1 Department of Chemical Engineering and Biotechnology, University of Cambridge, Pembroke Street, Cambridge, CB2 3RA, UK; 2 Department of Zoology, University of Cambridge, Downing Street Cambridge, CB2 3EJ, UK; 3 Department of Physiology, Development and Neuroscience, University of Cambridge, Downing Street, Cambridge, CB2 3DY, UK; 4 Center for Organismal Studies, University of Heidelberg, Im Neuenheimer Feld 329, 69120 Heidelberg, Germany

## Abstract

Light-sheet microscopy is an increasingly popular technique in the life sciences due to its fast 3D imaging capability of fluorescent samples with low photo toxicity compared to confocal methods. In this work we present a new, fast, flexible and simple to implement method to optimize the illumination light-sheet to the requirement at hand. A telescope composed of two electrically tuneable lenses enables us to define thickness and position of the light-sheet independently but accurately within milliseconds, and therefore optimize image quality of the features of interest interactively. We demonstrated the practical benefit of this technique by 1) assembling large field of views from tiled single exposure each with individually optimized illumination settings; 2) sculpting the light-sheet to trace complex sample shapes within single exposures. This technique proved compatible with confocal line scanning detection, further improving image contrast and resolution. Finally, we determined the effect of light-sheet optimization in the context of scattering tissue, devising procedures for balancing image quality, field of view and acquisition speed.

Light sheet fluorescence microscopy, LSFM, is revolutionising the way in which complex and living biological samples are imaged at high temporal resolution. The key principle is to illuminate the object at right angle to the observation axis with a thin sheet of light, thus defining an excitation plane that is imaged by a wide-field detector system^1^,^2^,^3^. The technique offers optical sectioning capability similar to confocal microscopy, but with two major advantages. Firstly, the sample is illuminated only in the focal plane of the detection objective, which dramatically increases signal to noise ratio and reduces photodosage and associated phototoxicity in the sample^4^. This is in stark contrast to confocal imaging, where the entire sampling volume is exposed to light when recording a single image plane. Secondly, because in LSFM both sample illumination and signal collection processes are parallelized, much higher acquisition rates are possible compared to single point scanning techniques and for example hundreds of frames per second are routinely achieved in LSFM. Therefore for fast volumetric imaging of complex organisms, light sheet microscopy is rapidly becoming the technique of choice for many scientific questions, e.g. in developmental biology^5^ and in the neurosciences^6^.

One of the key determinants of image quality in LSFM is the light-sheet itself. The thickness of the light-sheet defines the illuminated sample plane and thus determines both the axial resolution of the detected signal and, through rejection of background signal, also image contrast in the detection plane^7^. A highly focused light sheet thus results in optimal image quality. However, there is a finite spatial extent over which a tight focus can be maintained; for a Gaussian beam this is determined by the Rayleigh length. A highly focused beam features a correspondingly small Rayleigh length and this limits the field of view, over which good image quality is preserved. There is thus a trade-off between field of view, light sheet thickness, and image quality, and a flexible method to adjust the light sheet properties to suit different experimental requirements is highly desirable. For example, the tracking of cell division and movement requires the simultaneous recording of very large numbers of cells at relatively coarse resolution but over large field of views^8^. For the functional imaging of protein pathways, on the other hand, a subcellular image resolution is required and this necessitates the imaging over smaller field of views^9^. Some biological questions require the retrieval of data from deep within a sample (e.g. during organ development) and others are confined to regions closer to the surface (e.g. during observation of epithelial cells^10^), and the scattering properties may change dramatically between these applications or even as a function of imaging depth within the same sample. In short, to obtain optimal image quality it is essential that the illumination geometry is adapted to the problem at hand^11^,^12^,^13^.

In this paper we present a new approach to control light-sheet geometry through incorporation of a telescope built of two electrically tuneable lenses in the excitation path of a digitally scanned light sheet microscope (dSLM)^14^. Specifically, the system permits the rapid translation of the light-sheet focal waist along the illumination axis and adjustment of the light-sheet thickness through control of the numerical aperture, NA, of the illumination system. The light-sheet can thus be conveniently and dynamically adjusted to balance image quality, field of view, and acquisition speed to match the application requirement. For example, through acquisition of multiple images with the light-sheet focus translated across a sample and combination of the images, the field of view can be greatly expanded, if the increase in acquisition time can be tolerated. Alternatively, where speed is of essence, the focus of the beam can be translated in real time during single acquisition frames (20 ms) to track topological features of interest at high speed. We show how arbitrary light-sheet ‘shapes’ can be generated to match the object topology in 3 dimensions, retrieving optimal image quality with minimal acquisition time and light exposure.

Because the refractive power of the lenses is changed directly, no mechanical movement of lens position is required to adjust the light sheet properties, which improves stability, reproducibility and speed. We show that this makes the method perfect to be deployed in conjunction with the confocal slit scanning technique^15^,^16^, which involves the synchronised movement of an active pixel-line across a complementary metal-oxide-semiconductor (CMOS) chip with that of the laser beam creating the light sheet, and greatly improves image contrast by out of focus light rejection. Using the adaptive, digitally scanned beam described above it is possible to optimise light delivery into the region imaged by the confocal slit for all imaging conditions.

Finally, we show how image quality varies under different scattering and illumination conditions in representative biological samples and emphasize the importance of adapting light sheet properties for a given experimental environment. Our method is straightforward to implement in any existing light sheet microscope, including OpenSPIM^17^, and microscopes employing Bessel beam^18^ or multiphoton excitation^19^,^20^.

## Results

### Tuneable lens telescope

**Figure 1:**
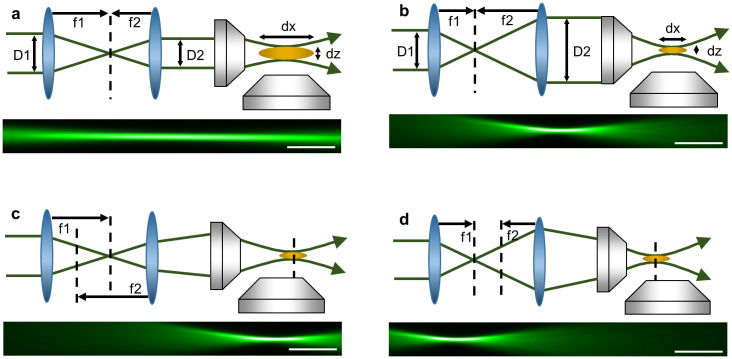
Principle of light sheet adjustment with tunable lenses. (a) and (b): diagrams explaining how variation of the tuneable lenses foci f1 and f2 changes the magnification, D2/D1, thus changing lateral (dx) and axial (dz) extent of the beam focus. Below are measured beam profiles for 0.13 NA and 0.3 NA illumination beams, respectively. (c) and (d) show how displacement of the foci f1, f2 changes beam convergence/divergence thus translating the illumination focus axially. Below are measured beams (0.3 NA) with positions displaced by 150 μm. All scale bars 30 μm.

An overview of the custom built dSLM used in this work is given in the Supplementary Fig. S1 (for detailed description see Methods section). A key feature of the system is a telescope featuring two electrically tuneable lenses through which the laser beam generating the light sheet is passed before entering the excitation objective. The telescope permits the continuous variation of the excitation beam diameter and divergence at the back aperture of the excitation objective, to result in corresponding changes of light-sheet thickness and position of the focal point. Changing the beam diameter at the back aperture of the illumination objective varies the numerical aperture, NA, in the excitation path and hence light sheet extension/thickness (see diagrams in Figure 1a, b). Changing the divergence mimics the quadratic defocus phase resulting in the translation of the beam focus (diagrams in Figure 1c, d).

In our setup the tuneable lenses were placed 175 mm apart to utilise the most dynamic part of the their focal range (50–125 mm), yielding a magnification in the range 0.4–2.5. Therefore, with a 0.3 NA excitation objective, the range of illumination NAs was variable from 0.05–0.3 corresponding to light-sheet thicknesses ranging from 10 to 1.5 μm (FWHM – Full Width Half Maximum) and extensions from 850–36 μm (FWHM – double Rayleigh range), respectively. In practice we restrict our NA to a ‘useful’ range from 0.13 (thickness 3.5 μm, extension 180 μm) to 0.30, (thickness 1.5 μm, extension 36 μm) to obtain optimal imaging conditions for samples presented here. Representative images are shown in Figure 1. For more information about the tuneable telescope design see Supplementary Discussion.

To determine the quality of this approach, we 1) measured the excitation beam profiles for different illumination NAs and positions in the camera field of view and compared those to theoretical profiles for Gaussian beams (Supplementary Fig. S2); 2) imaged fluorescent beads and measured their apparent extension along the detection axis as function of their position along the illumination axes (Supplementary Fig. S2). The results show that the generated beams are close approximations to ideal Gaussian beams with NA ranging from 0.13 to 0.3 across the entire camera field of view. To conclude, our telescope is a high quality, flexible, and low cost solution to adapt the illumination profile to achieve the desired light sheet geometry.

### Adjustable light sheet in slit-scanning mode

Because light-sheet microscopy uses wide-field detection, it is susceptible to image deterioration by scattering and this counter-balances the advantage of high acquisition speeds. However, a recently published method called slit-scanning offers an interesting solution to this problem^15^,^16^. The technique involves the scanning, in the detection path, of a slit, which is synchronized with the illumination beam as the latter is scanned across the sample. The slit, in practice an active pixel line on a scientific CMOS camera that is translated across the detector array with the other pixels rendered inactive, is arranged to be in the confocal plane of the excitation beam and hence rejects scattered light from out of focus locations. The main challenge for the method is the need for precise temporal and spatial synchronization between excitation beam and the slit. The tuneable lens telescope is perfectly adapted to this task: Contrary to mechanical solutions requiring the repositioning of lenses they can produce a fast and purely axial translation without lateral beam drift, which would prohibit the use of slit scanning. We show in the following that the beam focus can be repositioned at will and the light sheet adapted arbitrarily to the sample geometry, during live imaging with slit scanning, without any loss of synchronisation.

**Figure 2:**
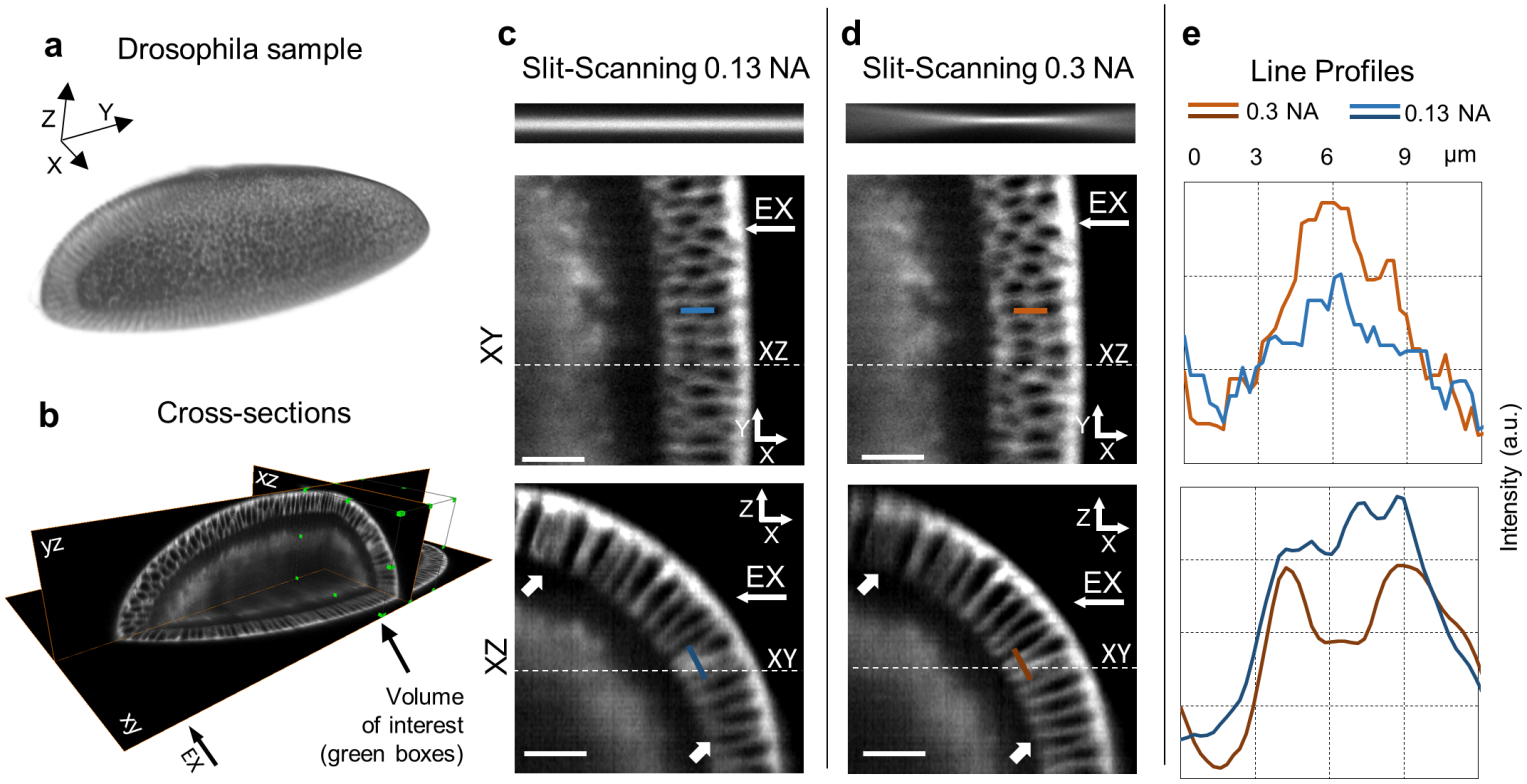
Image quality dependence on high and low illumination NA in slit-scanning. (a): a 3D rendering of membrane labelled (GFP), stage 5 Drosophila embryo. (b): XY, XZ and YZ cross-sections (indicated) through the sample in (a) with a green box constraining a volume of interest. (c) (d): XY (top) and XZ (bottom) cross-sections of volume of interest acquired in slit-scanning mode using lower NA (0.13) illumination for (c) and higher NA illumination (0.3) for (d). The beams are visualised above the panels. White arrows indicate areas of strongest difference in contrast and resolution between higher and lower NA illumination modes in XZ cross-sections due to bowtie effect (see main text). (e): profiles through equivalent membrane structures, indicated with color-coded lines in the sample-cross-sections in (c) and (d). The dashed white lines in panel (c) and (d) show relative positions of the XY and XZ cross-sections. All scale bars 15 μm. EX arrows indicate excitation beam direction.

Figure 2 demonstrates the benefits of combining our system with slit-scanning. A membrane labelled, stage 5 Drosophila embryo (see methods) is first illuminated with a 0.13 NA beam to obtain a low resolution preview of the entire sample in slit scanning mode. We repeated the acquisition with a light-sheet generated by a highly focused 0.3 NA illumination beam, repositioned near the centre of the volume of interest (green volume indicated in Figure 2b). No resynchronisation was necessary of the beam and the slit movements between the two measurements. The contrast is enhanced in the XY detection plane (Figure 2c, d, top row) in the region of the beam focus position. This can be visualized in the increased peak intensity in line profile through a cell membrane (Figure 2e, top row). The improvement in image quality is most pronounced in XZ cross-sections through the same volume, with cell membranes being much sharper. (Figure 2c, d, bottom row). The line profiles through two adjacent membranes (Figure 2e, bottom row) shows increased resolvability resulting from the use of a thinner light sheet. In the image obtained using the 0.3 NA light sheet there is a clear minimum between two membranes, which is missing from the image generated with the 0.13 NA light sheet.

### Trade-off between light sheet extension and thickness

A well-known limitation of digitally scanned light-sheet microscopy is the reduced field of view obtainable with highly focused light because of the ‘bow tie’ shape of a Gaussian beam. Image quality deteriorates rapidly in the wings of highly divergent, high NA beams (Figure 2d, XZ projection, white arrows and Supplementary Fig. S3). However, one can overcome the resulting problem of the limited useful field of view by acquiring multiple image frames, each obtained with the focal position of the light-sheet translated to a different position relative to the sample^11^,^12^. Images can then be stitched, or fused (see methods), to yield a compound image of higher resolution and contrast over larger field of views.

**Figure 3:**
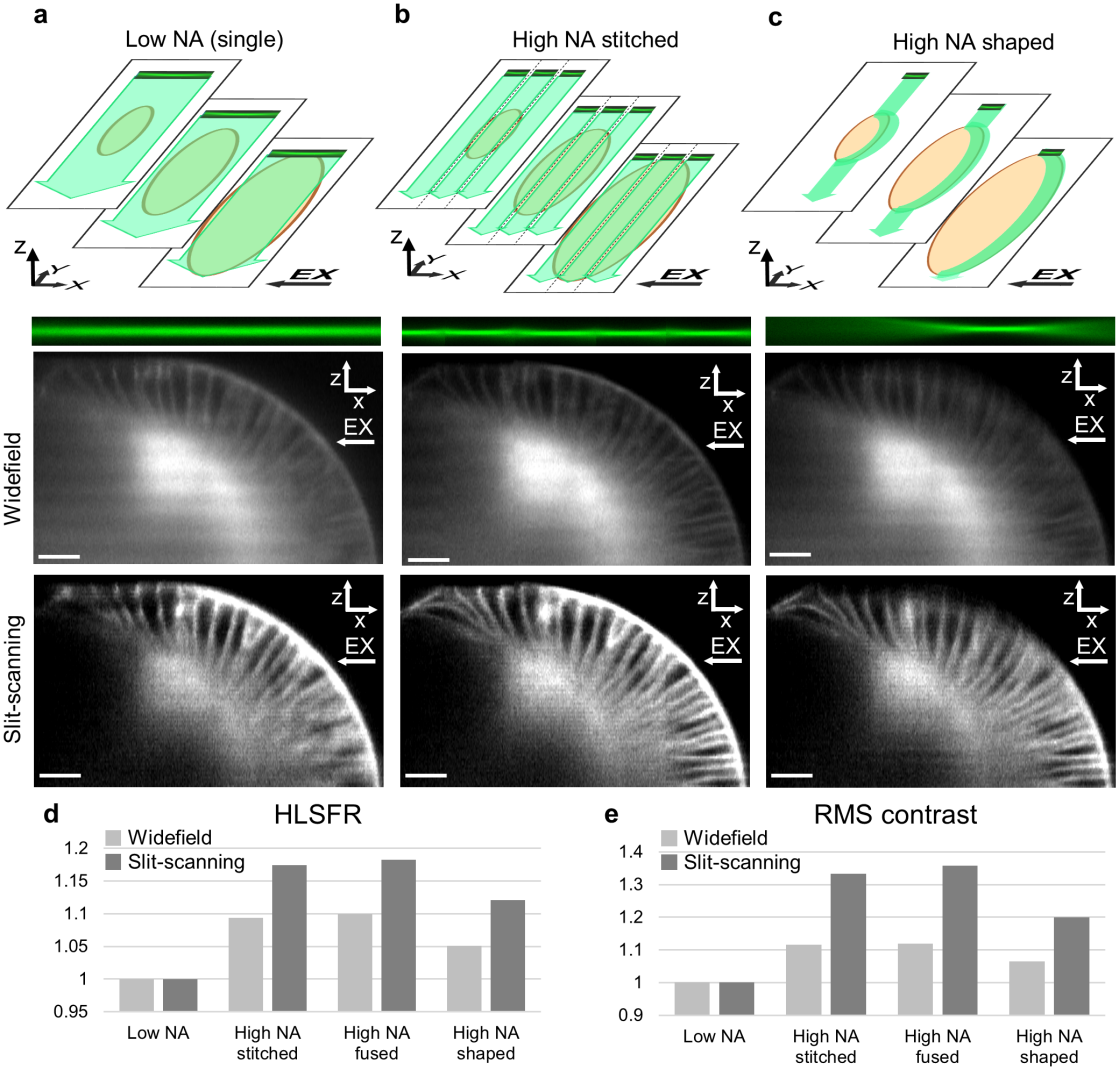
Image quality in different illumination modes. Top row depicts stack acquisition using 3 different illumination modes (see main text for details). Orange ellipse symbolizes a sample, green arrows depict scanning of an illumination beam focus to create a light sheet. (a): low NA single frame acquisition with large field of view. (b): high NA illumination axially translated to capture multiple images and combine them using simple stitching into compound image with large field of view. (c): high NA illumination shaped to match volume of interest. Middle and bottom row show XZ cross-sections of volumes acquired using wide-field mode and in slit-scanning mode, respectively. The sample is the membrane labelled (GFP), stage 6 *Drosophila* embryo. (d) (e): graphs quantifying differences in image resolution (HLFSR) and contrast (RMS contrast), see methods. The values are normalized in respect to low NA mode, showing relative improvements of using high NA modes. All scale bars 20 μm. EX arrows indicate excitation beam direction.

Our system is compatible with such an acquisition mode and eliminates the need for repositioning the specimen. We imaged another membrane labelled, stage 6 Drosophila embryo, using five repositioned NA = 0.3 beams, each featuring a field of view of 36 μm, to cover 180 μm – the diameter of the Drosophila embryo (Figure 3b, Supplementary Fig. S3). The images were combined with two different methods, by a simple image stitching algorithm (Figure 3b) or by a wavelet fusion method (Supplementary Fig. S3, see Methods section for details) a discussion and comparison of the quality of the different acquisition modes possible with the present instrument is part of a later section in this article.

Here, we note that the tuneable lens method is ideally matched to such acquisition strategies (focus translation) due to the inherent high stability and reproducibility in repositioning the beam. Multiple stacks could be acquired over long periods of time (e.g. 8 hours) with insignificant drift along any of X, Y or Z, which greatly simplified the stitching/fusion of the image data. Moreover, the fast response of the lenses allowed repositioning the beam and acquiring images for each Z plane as opposed to translating the beam after each stack. This can reduce acquisition delays and thus motion artefacts due to sample distortion or movement and improve the quality of the stitching/fusion process (Supplementary Fig. S4).

Naturally, this method slows down acquisition speed, but the rapid response time of the tuneable lens system on the one hand, and the inherently high speed of the light sheet technique on the other^16^, permitted the acquisition of a full volume stack containing 100 slices, each measuring 700 × 2048 pixels, in 11 s (2.2 s for single, non-stitched, stack, with 20 ms exposure time per frame). Such speeds are acceptable for the study of many dynamic processes and are at least an order of magnitude faster than typical commercial confocal microscopes. Similarly, the need for multiple acquisitions increases the photo-dosage in the sample, however, because of the inherent light efficiency of light-sheet microscopy the overall exposure of the sample is still considerably lower than in confocal microscopy^21^. For example using stacks of 100 images each point is illuminated 100 times in confocal microscopy, whereas for the given example in the LSFM case the increase is only a factor of 5.

### Light-sheet sculpting

Another way of circumventing the usual trade-off between resolution and field of view is possible with rapidly tuneable lenses: the ‘live sculpting’ of the light sheet to outline the shape of the sample. For example, epithelial samples like early stage *Drosophila* and zebrafish embryos contain features of interest (cells) only in a thin outer layer of their ellipsoidal or spherical shape, respectively. Normally, to image such samples using high NA illumination would require acquisition and stitching/fusing of multiple stacks (as outlined in the previous paragraph). Here, however, it is possible to shape the high NA light-sheet to match the features of interest and we show how it is thus possible to obtain, with acquisition of only one stack, similar image quality as with multiple combined stacks.

Through computer control of the signals fed to the electrically tuneable lenses it was possible to vary the illumination beam position during individual acquisition frames with exposures of 20 ms (see Supplementary Fig. S5, 6). This permitted us to generate a light sheet such that the focal waist in each XY traced out characteristic features in the corresponding plane of a 3D sample (Supplementary Fig. S7 and Supplementary Movies 1–3). Slicing the volume with sculpted light sheets thus permits to track arbitrary sample topologies, in our case the epidermis of a *Drosophila* embryo (labelled with membrane-targeted GFP, Figure 3c). By adjusting the light sheet ellipse for each slice in the stack we created a quarter ellipsoid shell as an approximation to the epidermal outline of the sample.

Other techniques, like mechanical zoom lenses^13^ or spatial light modulators, which feature response times in the order of tens of milliseconds, are not fast enough to generate gradients and curvatures in light sheets without sacrifices in acquisition speed. The limiting factor here is the slew rate of lens focus changes which limits the repositioning speed of the light sheet and was found to be 15.3 μm/ms in our system (with 0.3 NA illumination). This determines the maximum achievable gradient in the light sheet topology over the field of view. For example, during a 20 ms exposure it was possible to translate the focus linearly over 306 μm. A full characterisation of the lens response and is given in the Supplementary Discussion and Supplementary Fig. S5, 6.

### Comparison of image quality between different imaging modes

The tuneable lenses therefore give the user a choice between four modes of imaging: low NA illumination for large field of view and fast acquisition (Figure 3a and 2c); high NA illumination for small field of view, fast acquisition times and superior image quality (Figure 2d); multiple high NA acquisitions for large field of view, superior image quality but decreased acquisition rates and increased light exposure (Figure 3b); High NA shaped illumination matched to the sample topology for superior image quality and fast acquisition times (Figure 3c).

The comparison of image quality obtainable with these imaging modes is presented in Figure 3, which shows similar Drosophila embryo and field of view as shown in Figure 2. Visual inspection confirms that the high NA illumination images exhibit superior quality compared to the low NA illumination modes. Most importantly however, the high NA shaped light sheet mode shows considerable improvement in resolution and contrast, similar to that of stitched or fused high NA mode, but without the sacrifice in acquisition frame rate. These findings are quantified by using two image quality metrics: RMS contrast^22^ (see methods) and relative weight of the Fourier components of the images^23^ - ie High-to-Low-Spatial-Frequency-Ratio (HLSFR - see methods) presented in Figure 3d, e. This data shows that the benefit of using higher NA beam is more pronounced in slit-scanning mode (18% and 35% improvement in HLSFR and RMS contrast for slit-scanning as opposed to 9% and 11% for wide-field when using higher NA illumination stitched mode over low NA). This finding emphasize further the importance of compatibility between tuneable lenses and slit-scanning.

### Effects of scattering on high NA illumination

**Figure 4:**
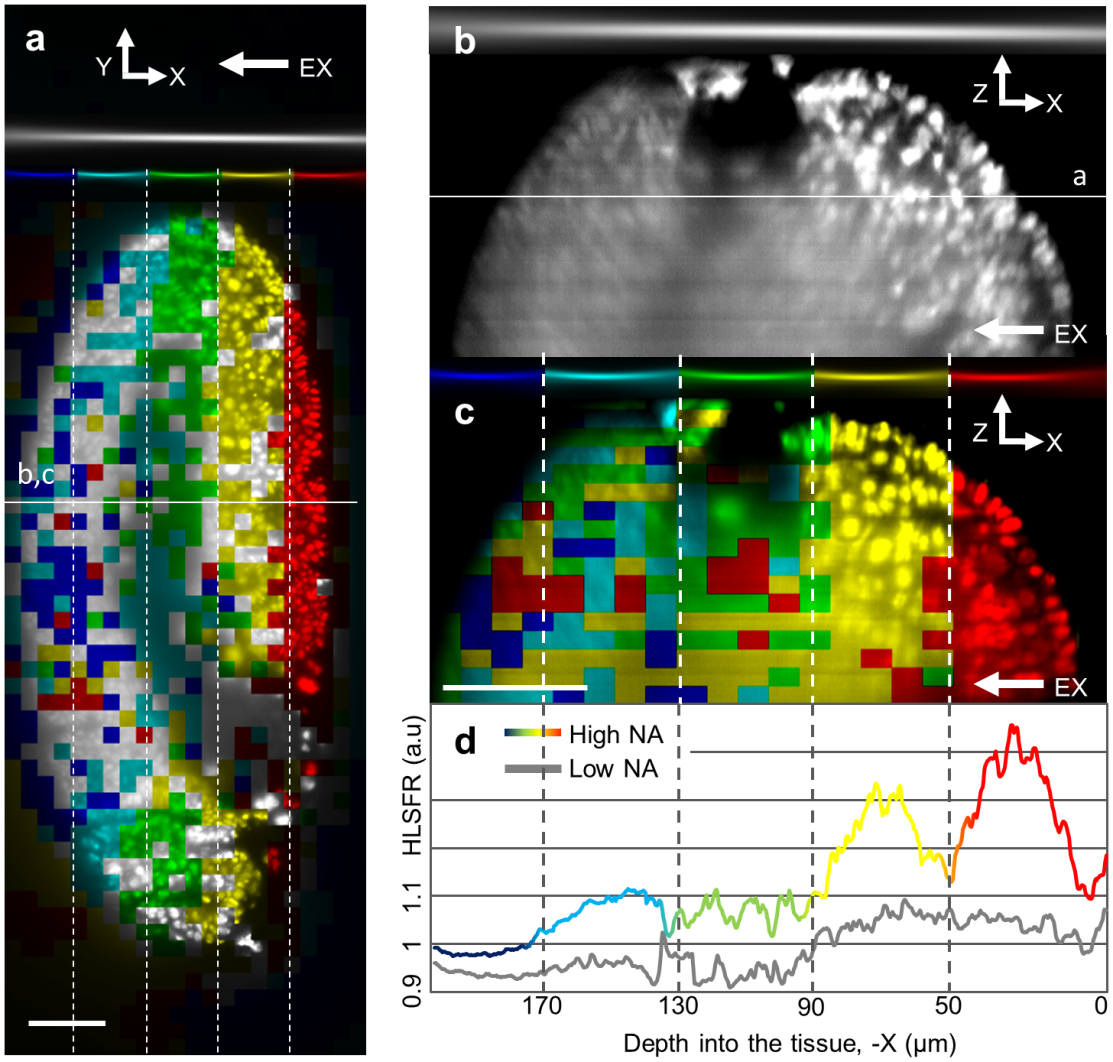
Scattering analysis using color-coded contrast variations through a sample (late stage Drosophila embryo with GFP labelled nuclei). (a): XY cross section through a stack combined from low (0.13) and high (0.3) NA images based on local highest contrast (See main text). Above the excitation beams used to obtain the image color-coded to visualize which raw data stack a given part of the compound image comes from. (b): XZ cross-section through only the data obtained using low NA illumination. (c): XZ cross-section through compound image obtained using high NA illumination data only. (d): resolution measure (HLSFR) for each YZ plane (perpendicular to illumination) along the illumination direction into the sample (-X axis). Rainbow colour ‘High NA’ line corresponds to a compound image created from only high NA beams (c) while ‘Low NA’ line is from a raw stack obtained using low NA illumination (b). All scale bars 40 μm. EX arrows indicate excitation beam direction.

The benefits of strong focusing of the illumination sheet into the sample for high NA illumination is ultimately limited by scattering. Complex biological samples like Drosophila or zebrafish embryos, scatter and distort the illumination light, making it impossible to maintain a tight laser focus at increasing penetration depth. We assessed this by comparing the image quality obtained in stage 10 *Drosophila* embryos with more developed mesodermal tissue for NA = 0.13 and NA = 0.3 beams, the latter translated axially to 5 different positions to cover the same field of view. We analysed local contrast by dividing the six resulting stacks into sub-volumes measuring 8 × 8 × 5 μm^3^ and determined RMS contrast for each one (for details refer to methods). To visualize the results we selected the boxes (subvolumes) with highest contrast and combined them into a single stack. The result is shown in Figure 4a, which is an XY cross-section from this stack. Near the top of the image, in grey scale, is shown the beam profile of the NA = 0.13 beam. Below the positions of the 5 axially displaced beam foci for the NA = 0.3 beam are shown in different colour. Six stacks were thus taken in total. The boxes (subvolumes) noted above were colour coded depending on which stack they were taken from (e.g. grey indicates the data was taken from the stack with the 0.13 NA beam, red corresponds to the stack with the 0.3 NA beam focus at the right most position, etc.). Figure 4a reveals that at up to 90 μm penetration depth there is good correspondence between the box colour patterns and corresponding beam focus positions, indicating benefits from high NA illumination. Deeper into the tissue however the colour pattern is lost and the box pattern contains many panels from the low NA illumination indicating that no benefit is gained from high NA illumination. Figure 4b–d quantifies this observation. Using the same raw data (but presented as XZ cross-sections) we created two separate stacks: one obtained using a single 0.13 NA illumination (Figure 4b) and one colour-coded using five 0.3 NA beams (Figure 4c). A Fourier analysis (HLSFR – see Methods) was performed for both stacks to quantify image quality in each YZ plane (perpendicular to the displayed XZ cross-sections) and plotted below. Clearly there is considerable improvement (up to 50%) for the first 90 μm of the higher NA (colour line) illumination over the lower NA (grey line) case but the advantages diminish for deeper penetration depth, consistent with the visual observation in Figure 4a. For the depicted example it is thus sufficient to stitch stacks obtained from 2 high NA beams and 1 from a low NA beam to achieve the same image quality as stitching data from 5 high NA beams. This finding confirms the relevance of optimizing light sheet conditions for the required field of view, image quality and imaging speed. The graph in Figure 4d also indicates a very strong coupling between beam thickness and image quality, with red and yellow peaks matching the respective beam thicknesses. Here, further improvements may be possible by positioning the beam waists at positions intermediate to those shown, i.e. displacements over less than 2 Rayleigh ranges. Naturally this comes with increases in acquisition time.

In Figure 4c (XZ cross-section) the colour pattern is also lost deeper along the detection path (-Z direction in the image). This is due to scattering effects along the detection axis and indicates that the light sheet conditions need to be optimized also according to the depth of penetration into the sample (i.e. there is no need to use high NA beam, which extends acquisition time, deeper into the stack as the benefits are lost due to scattering in the detection path). This effect is better visualised in the Supplementary Fig. S8, where instead of dividing the stacks into 3D boxes we divided it into 2D panels (XY plane) making the pattern more variable along detection direction.

### Shadowing effects

**Figure 5:**
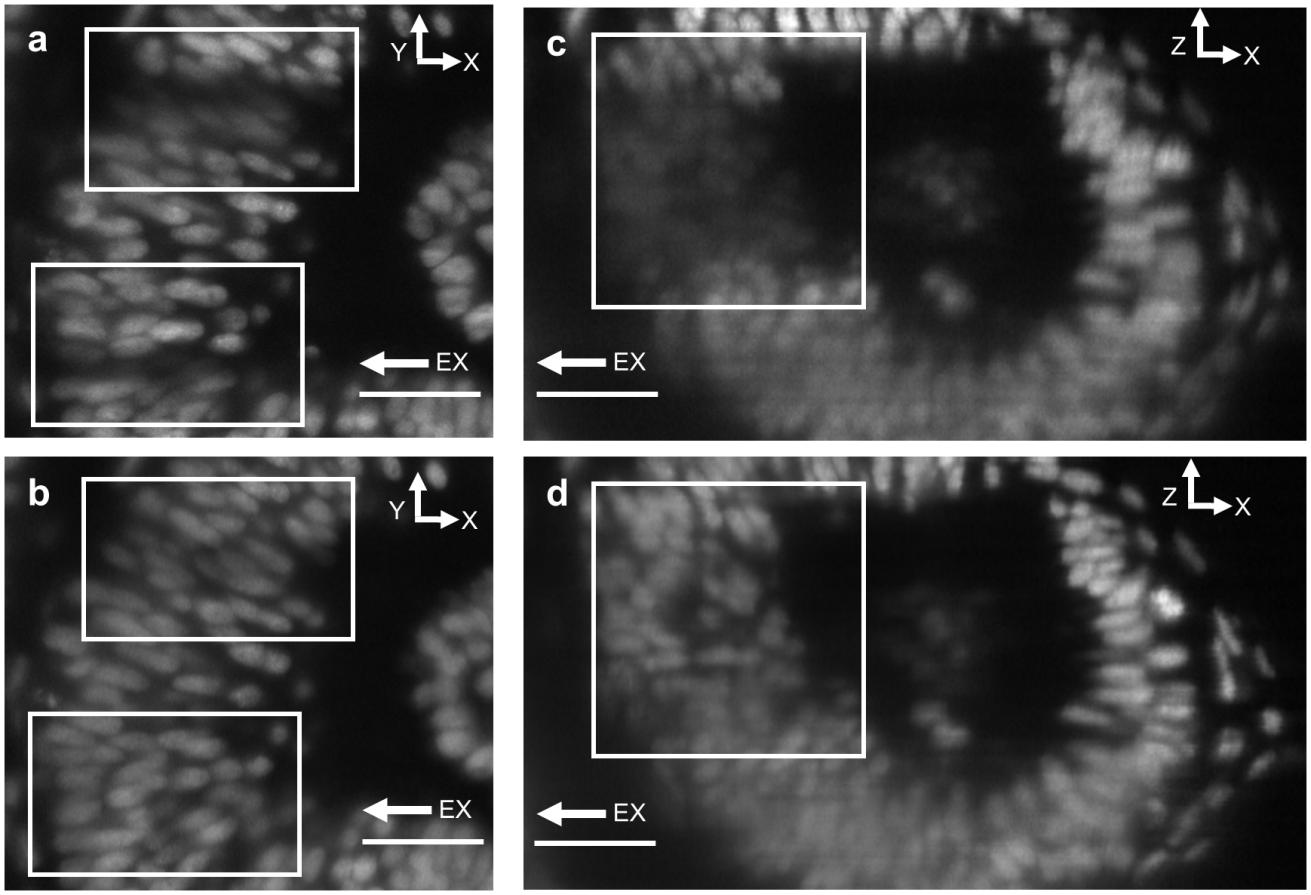
Eye of a 24 hpf Zebrafish embryo with GFP labelled nuclei – a zoom into the retina behind the lens. (a), (b): XY cross-section of the same volume acquired with low NA and high NA stitched using ImageJ respectively. Boxes in panel (a) highlight areas where shadows appear due to excitation beam being highly scattered by the eye lens. In images (b) and (d) this effect is minimized by using higher NA excitation (see main text). (c), (d): XZ cross-sections of low NA and high NA (ImageJ stitching) volumes respectively. The shadowing artefacts are highlighted with a box in image (c), while image (d), acquired with high NA excitation, shows improvement in the same area. All scale bars 30 μm. EX arrows indicate excitation beam direction.

There can be benefits, however, to using high NA illumination at depth in some samples, where diffuse light scattering is less of an issue than so called shadowing, a common problem in LSFM. The latter is caused by strong refractive index changes locally in a sample. When illumination light passes through, it becomes deflected and thus vanishes behind the causing features. The most commonly used approach to address this problem is to pivot the light sheet in its plane^24^ and thus illuminate at different input angles to average out the effects of scattering features. Using a high NA beam can offer analogous advantages as shown in Figure 5, where the edge of a lens in zebrafish embryo eye creates strong shadows in low NA illumination, an effect that is much less pronounced for the high NA illumination case.

## Discussion

The optimization of illumination condition is crucial for obtaining the highest image quality in light sheet microscopy^11^,^12^,^13^. Our method of using tuneable lenses in the excitation path provides many new possibilities and advantages over existing techniques.

The foremost benefit of the tuneable lenses is their speed. This had been previously used for fast z-scanning in microscopy^25^. Here we show the advantages of optimising illumination conditions in light-sheet microscopy. The adjustment of light-sheet geometry can be performed orders of magnitude faster than using standard lenses, which are mechanically translated, commonly known as ‘zoom lens’ configurations^13^. Both light sheet thickness and axial position of the focal waist of the beam can be rapidly adjusted, eliminating the need to reposition the sample to match the area of interest and offering simplicity and stability in imaging. The translation also enables acquiring larger fields of view with high NA excitation by acquiring multiple stacks with different light sheet positions and combining these stacks into one. This approach is faster and more stable over long periods of imaging than moving a sample through a stationary high NA light sheet^11^,^12^. Also, due to the speed of the lenses it is possible to acquire multiple images with translated focus for each slice in the stack. In such, the time delay between images to combine is greatly reduced.

We have also shown that optimized light sheet is even more important in the slit-scanning mode, where a thinner light sheet can yield relatively higher improvements than in wide-field mode making tuneable lenses an attractive addition to slit-scanning dSLMs.

Many biological samples in developmental biology require a large field of view (e.g. *Drosophila*, zebrafish), while having convex outer surfaces. As the tuneable lens system allows the light sheet to precisely lock onto to the outer surface, the light dosage for a given image quality is sharply reduced compared to multiple exposures at different relative displacements. This original approach to samples with complex topology is only achievable with tuneable lenses due to their speed and stability.

There are also other techniques used to overcome the trade-off between Gaussian light sheet thickness and length, for example Bessel beams^18^. This solution can offer superior image quality and is less susceptible to scattering but requires multiphoton excitation^19^,^20^, slit-scanning^23^ or complex phase adjustment^26^,^27^ to achieve its full potential. Tuneable lenses are considerably simpler to implement and compatible with many existing designs (OpenSPIM^14^, dSLM^17^, etc), including the Bessel beam setups. Combining tuneable lenses with Bessel excitation can offer higher flexibility and speed in choosing the field of view (Supplementary Fig. S9–10)^28^. Such a solution would also be capable of creating shaped Bessel beam light-sheets. Another technique involves the use of a Tunable Acoustic Gradient (TAG) lens to oscillate the excitation spot of a multiphoton light sheet at speeds compatible with beam scanning, such that the light sheet thickness and extension become decoupled^29^. The approach does not compromise acquisition speed but, is only valid for multiphoton excitation. In the linear excitation regime prohibitive background noise is generated by the ‘bowties’ of the scanning beam.

In summary, we have demonstrated a method of digitally scanned light sheet microscopy using electrically tuneable lenses to laterally shape, and axially translate, the excitation focus in order to achieve optimal illumination conditions for complex biological samples. The system presented here enables modulation of the focal position of the excitation beam during a single acquisition frame. This makes it possible to shape the intensity distribution of the light sheet to be optimal for the sample of interest. We highlighted the importance of choosing optimal illumination conditions for different scattering conditions and how our technique can be used to achieve this.

Future implementations and variation of the current ideas may include the use of spatial light modulators for Bessel beams and to shape the illumination beam: adaptive optimisation of image contrast would permit beam focus to be retained deeper into the sample, extending the advantages of high NA illumination and stitching. The tuneable lenses are also compatible with multiphoton illumination, having over 96% transmission in the NIR range and introducing less dispersion than equivalent zoom systems.

Multiview light-sheet microscopes could also benefit from tuneable lenses, regardless if the multiview is achieved by rotating the sample^24^, swapping the excitation and detection axes (iSPIM^30^) or using two detection and excitation objectives^31^,^32^. If a sample doesn't have a rotational symmetry, viewing it from different angles will result in the need for different field of views and hence different light sheet extensions. The tuneable lenses would allow the seamless switching between optimal light sheets for every view.

A final idea currently investigated is to automatically track the sample shape and adjust the light sheet to match it in order to achieve optimal image quality in samples strongly variable in time. This could then be applied to particle tracking, where a thin light sheet would follow a moving feature to generate best image quality without problems of a small field of view.

## Methods

### Ethics Statement

All animal work was approved by Local Ethical Review Committee at the University of Cambridge and performed according to the protocols of UK Home Office license PPL 80/2198.

### Samples

Aqueous solutions were prepared containing 1 μM of Rhodamine 6G (Sigma Aldrich) or FITC (Sigma Aldrich). Bead test samples were prepared using agarose (UltraPure™ Agarose, Sigma Aldrich) with a concentration of 1 g agarose per 100 ml dionised water, according to manufacturer's instructions. An aqueous solution containing fluorescent beads (FluoSpheres® Carboxylate-Modified Microspheres, 0.1 μm, yellow-green fluorescent, 505 nm excitation and 515 nm emission maxima, respectively, purchased from Life Technologies) was mixed into the agarose solution at 95°C at a volumetric concentration of 10^−6^ and continuously stirred to avoid aggregation of beads whilst the gel was left to cool down to room temperature for setting.

The *Drosophila* lines used was His2AvDGFP and SpiderGFP, which label all nuclei with GFP and all membranes with GFP, respectively^33^. The embryo preparation was performed as described in *Wieschaus* E et al. (1986)^34^. The embryos were mounted in a custom made dish (see microscope setup) using double sided adhesive tape. The dish was filled with PBS.

The detailed preparation of zebrafish embryos is described in He J et al. (2012)^35^. H2B-GFP transgenic zebrafish embryos were raised at 28.5°C and staged in hours post fertilization (hpf). Embryos were treated with 0.003% phenylthiourea (PTU, Sigma) at 8 hpf to delay pigmentation and were anaesthetised with 0.04% MS-222 (Sigma) prior to live imaging. At 24 hpf, the embryos were dechorionated and embedded in 1% low melting agarose in a custom-made imaging dish (see microscope setup for further details). The dish was then filled with imaging medium (1× Steinberg, 0.04% MS222 and 0.003% PTU).

### Microscope setup

A variant of a Digitally Scanned Light-sheet Microscope (dSLM) was developed specifically for the present work. Briefly, the light sheet was formed by scanning a laser beam (Argon Ion laser, Melles Griot) laterally through the sample via a custom designed beam scanning unit (SU) as shown in Supplementary Fig. S1. The SU comprised galvanometric scanning mirrors (6210H with 5 mm mirror, Cambridge Technology) and a telescope with a 60 mm f-theta lens (S4LFT0061/065, Sill) and 200 mm achromatic tube lens. The excitation beam was passed through a water-dipping excitation objective (Nikon 10×, 0.3 NA), marked EO in Supplementary Fig. S1. The illumination numerical aperture, NA, was continuously adjusted from 0.13 to 0.3 by controlling the input beam diameter at objective input aperture using an electrically tuneable telescope (built of two EL-10-30, Optotune; for a full discussion of the tuneable lens system, TL, refer to the Results section). The signals were collected at right angles to the illumination sheet via a water-dipping detection objective, DO (Nikon 25×, 1.1 NA). Both EO and DO were chosen in efforts to optimise image resolution given the constraints imposed by the imaging geometry. Excitation and collection objectives were mounted at 45 degrees from the vertical to permit imaging of large samples contained in horizontally placed Petri dishes or specimen chambers. Signals were recorded on a scientific CMOS camera (Hamamatsu Orca Flash 4.0 V2) to yield a field of view (field of view) of 532 by 532 μm^2^ and each camera pixel corresponded to an area of (260 nm)^2^ in the sample. For excitation, a 488 nm argon-ion laser was used. Its output was coupled into a single mode fibre to homogenise the beam profile prior to free space delivery into the microscope frame, and light power was continuously adjustable via an acousto-optical tuneable filter (AOTF, AA optoelectronics).

The Hamamatsu camera, which has a rolling shutter as opposed to global shutter, has the option of controlling the speed and delay of a activation and readout shutters rolling through the chip. This allows for creation of a virtual slit of active pixels between these shutters, which scan through the chip. By synchronising the light sheet scanning galvo mirror with the slit using Labview we obtained the confocal slit scanning mode. Imaging conditions for each figure, including slit-scanning settings, are given in Supplementary Table S1.

We define coordinate system such that we denote the XY plane to be that containing the light sheet, whilst the z axis is the optical axis of a the detection objective (consistent with nomenclature used in standard wide-field imaging microscopes). The illumination path is along –X, i.e. from the right hand side in all presented images.

### Data analysis

We used and implemented commonly available algorithms to analyse the image data. All the images were displayed with linear contrast curve and oversaturated to appear clearer in print. Typically we set the contrast line to saturate 1% pixels at each end of histogram. For comparing various modes of illumination the contrast was set for lowest NA illumination and the settings were propagated to higher NA images.

For image stitching we used the ImageJ ‘Pairwise Stitching’ plugin^36^. Briefly, that algorithm is used to match spatially common regions between two images by shifting the images and positioning them optimally. The overlapping regions in the resulting stitched image are seamlessly fused using a linear blending algorithm. In our work we used this method to stitch cropped regions from multiple images of the same object. For example, we typically recorded 5 images per sample plane, for which image quality varied across the field of view. We then stitched the images in a ‘stripe-by-stripe’ fashion (see also Results section) to combine those parts from each individual image for which contrast and resolution were highest.

We have also implemented a wavelet fusion algorithm building on native Matlab functions (Available on request from corresponding authors). Wavelet fusion has an advantage of recovering information from all the images as opposed to stitching were suboptimal parts of image are discarded.

To quantitatively compare images we calculated the spatial Fourier transform for each normalized image (Equation 1) and obtained 1D spectra against radii from the peak by summing spatial frequencies along rings corresponding to radii (Equation 2, 3)^23^.
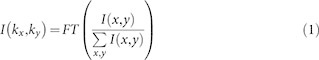

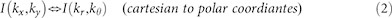

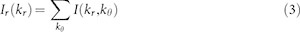


We defined a characteristic spatial frequency k_0_ as 0.2 μm^−1^, equivalent to a spatial feature of 5 μm dimension, which is the approximate size of the nuclei imaged in the embryos of both *Drosophila* and zebrafish. For each spectrum we then defined a low frequency region that for which k < k_0_ and a high frequency region as that for which k > k_0_. We integrated amplitudes in each region and took their ratio (HLSFR – High to Low Spatial Frequencies Ratio) as a relative resolution and contrast indicator (Equation 4).
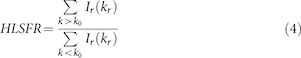


The other comparison criterion was RMS contrast^22^, which is defined as geometrical mean of all pixel intensities after subtracting the mean of these intensities (Equation 5).
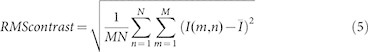
M and N are image width and height in pixels.

## Author Contributions

A.K.C., P.M., A.K., J.F.E. and C.F.K. conceived the project. A.K.C., A.K., P.M., J.F.E. and C.F.K. wrote the manuscript. A.K.C. prepared the figures (A.K. prepared Supplementary Fig. S2). The instrumentation was built by A.K.C., M.T.W. and J.F.E.. The control software was written by M.T.W., A.K.C. and J.F.E.. The advance image analysis (wavelet fusion) was performed by L.M.. The samples were prepared by A.K.C. A.K.C. and A.K. acquired the data. C.F.K. and J.F.E. supervised the project. All the authors reviewed the manuscript. J.F.E. and C.F.K. are equal senior co-authors.

## Supplementary Material

Supplementary Information Supplementary Movie 1

Supplementary Information Supplementary Movie 2

Supplementary Information Supplementary Movie 3

Supplementary Information Supplementary Information
