# Supplementary material for: Fast imaging of live organisms with sculpted light sheets
Source: Sci Rep. 2015 Apr 20;5:9385. doi: 10.1038/srep09385 (PMC4403519; doi:10.1038/srep09385)
Supplement: Supplementary Information [file srep09385-s4.pdf]

Supplementary Information

Fast imaging of live organisms with sculpted light sheets

Aleksander K Chmielewski, Anders Kyrsting, Pierre Mahou, Matthew T Wayland,
Leila Muresan, Jan Felix Evers, Clemens F Kaminski

Supplementary Discussion

Effects of spherical defocusing on beam quality. We analysed the effects of spherical phase defocusing on both Gaussian and Bessel beams by implementing simulations as detailed in Leutenegger M. et al (2006) . Supplementary Figure S9 demonstrates aberrations in both Bessel and Gaussian beams when using spherical defocussing. These aberrations deteriorate the beams particularly along the axial dimension, but showing a much lesser effect on radial quality (compare graphs in Supplementary Fig. S9). Spherical defocusing therefore affects light sheet thickness only minimally. Indeed, we did not detect any deterioration of image quality in light sheet microscopy when translating the illumination axially with tuneable lenses (Supplementary Figure 2A). We found that both beam length (FWHM) and thicknesses (σ) are in accordance with the theoretical parameters of a Gaussian beam, indicating a lack of major aberrations.

Interestingly, our simulations show that bessel beams are less affected by spherical defocussing. This is due to different intensity distributions in the back aperture of the illumination objective (Supplementary Fig. 10). The intensity profile required to generate a Bessel beam is a thin ring as opposed to a radial Gaussian in case of a Gaussian beam. As such, variation in phase does not feature as strongly in Bessel beam illumination. This observation confirms attractiveness of tuneable lenses for use with Bessel beams.

Tuneable lenses control. The tuneable lenses have a well documented by manufacturer nonlinear response of the focal length to the driving current signal. It is therefore possible, with the knowledge of the excitation path optics, to devise a function describing relation between a desired position of a light-sheet in the camera field of view and required current signal. In this work however we obtained this function from calibration. We positioned multiple light-sheets across camera field of view obtaining corresponding control signals and linearly interpolated them to create a continuous function linking current signal and light-sheet position. We found this approach insusceptible to temperature variations.

Such calibration is sufficient for imaging with low acquisition rates (below 5 Hz). For higher acquisition rates, especially in case of light-sheet sculpting, the lenses operate in their transient rather than steady state response. Supplementary Figure S5 shows this transient response to a step signal. In fact we used a sigmoid function as the lenses create artefacts in light-sheet when driven with discontinuous signals (see insets in Supplementary Fig. S5). This response allowed us to calculate the slew rate of the lenses, which is different for focusing (15 µm/ms) and defocusing (13 µm/ms).

To generate desired light-sheet shape during fast acquisitions we devised an algorithm to correct control signal. It differentiates the desired signal (after corrections resulting from steady state calibration of non-linear response) and multiply the result by a correction factor (larger than 1). Because of the unsymmetrical step response the correction factor for negative derivatives must be larger than for positive ones (see equations in Supplementary Fig. 6) . Choosing these factors requires a calibration for each desired light-sheet. Supplementary Figure S6 shows sinusoidal light-sheet generated with uncorrected and corrected signals and how the control signals look like before and after correction.

Supplementary Table S1. Imaging conditions

Fig.	Panels	Global expo-sure (ms)	Pixel exposure (ms)	Slit width		Beam thickness FWHM (µm)	Excitation NA	Laser power 488nm (µW)
				pix	µm*			
2	(c)	20	0.2	20	5.3	3.5	0.13	300
	(d)	20	0.1	10	2.6	1.6	0.3	300
3	(a), widefield	20	N/A	N/A	N/A	3.5	0.13	300
	(a), slit-scanning	20	0.2	20	5.3	3.5	0.13	300
	(b)(c), widefield	5*20	N/A	N/A	N/A	1.6	0.3	300
	(b)(c), slit-scanning	5*20	0.1	10	2.6	1.6	0.3	300
	(d)(e), widefield	20	N/A	N/A	N/A	1.6	0.3	300
	(d)(e), slit-scanning	20	0.1	10	2.6	1.6	0.3	300
4	Coloured parts, (c)	5*100	N/A	N/A	N/A	1.6	0.3	100
	Grayscale parts, (b)	100	N/A	N/A	N/A	3.5	0.13	100
S3	(a)	50	N/A	N/A	N/A	3.5	0.3	300
	(b)	50	N/A	N/A	N/A	1.6	0.13	300
	(c)	100	N/A	N/A	N/A	3.5	0.3	100
	(d)	100	N/A	N/A	N/A	1.6	0.13	100
S7	all	20	0.1	10	2.6	1.8	0.25	600
S8	all	5*100	N/A	N/A	N/A	1.6	0.3	100

* in object plane

Supplementary Figure S1. Detailed optical setup

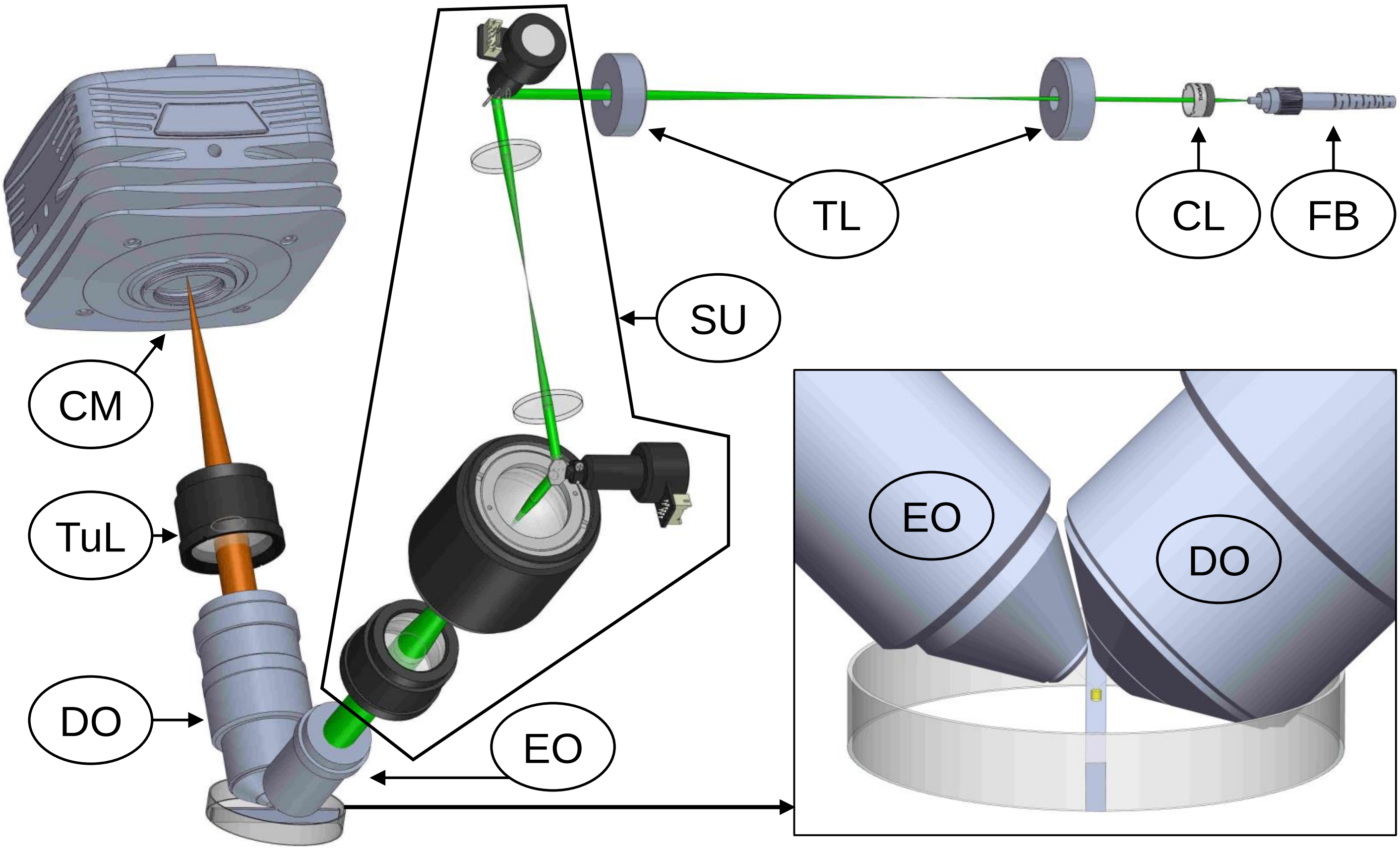

Supplementary Figure S1. Detailed optical setup. Light from 488 Argon Ion laser (Melles Griot) is passed through an AOTF (AAoptoelectronics) and fibre-coupled into single mode fibre (multiple laser kineFLEX, qioptiq). The output (FB) is collimated using achromatic lens (CL)(Thorlabs) to achieve 1.6mm beam diameter. It is aligned into the Thorlabs cage system containing the rest of the optics using dielectric mirrors (Thorlabs BB1E02) on kinematic mounts (Thorlabs). The tuneable lenses (TL)(Optotune EL10-30) being 175mm apart give the magnification range of 0.4 to 2.5. The tuneable telescope is kept very close (25mm) to the first galvo mirror to avoid overfilling the mirror due to divergence (which is necessary to axially translate the focus of the excitation objective (EO)). The relay telescope ensures the excitation beam angle doesn't change during scan (by ensuring that only beam angle and not position changes at the back focal plane of telecentric lens and excitation objective). A second galvo mirror is positioned at the back focal plane of telecentric lens (Sill, 60.5mm effective focal length), which creates a telescope with 200mm achromatic lens (Thorlabs). The galvos, scan lens and tube lens constitute scanning unit (SU). A second pair of steering mirrors corrects for any misalignment in the sage system and permits fine tuning of the angle of the excitation beam to match detection plane. The image is collected using Nikon 25X, 1.1NA water-dipping detection objective (DO) mounted on a piezo stage (PI, PIFOC 726). The movement of the piezo is synchronized with galvo mirrors using DAQ card (NI, PCIe 6323). The collected light is filtered (Semrock) and passed through 200mm Nikon tube lens (TuL) to form an image on the sCMOS camera (Hamamatsu Orca flash 4.0 V2).

Supplementary Figure S2. Analysis of illumination beam quality

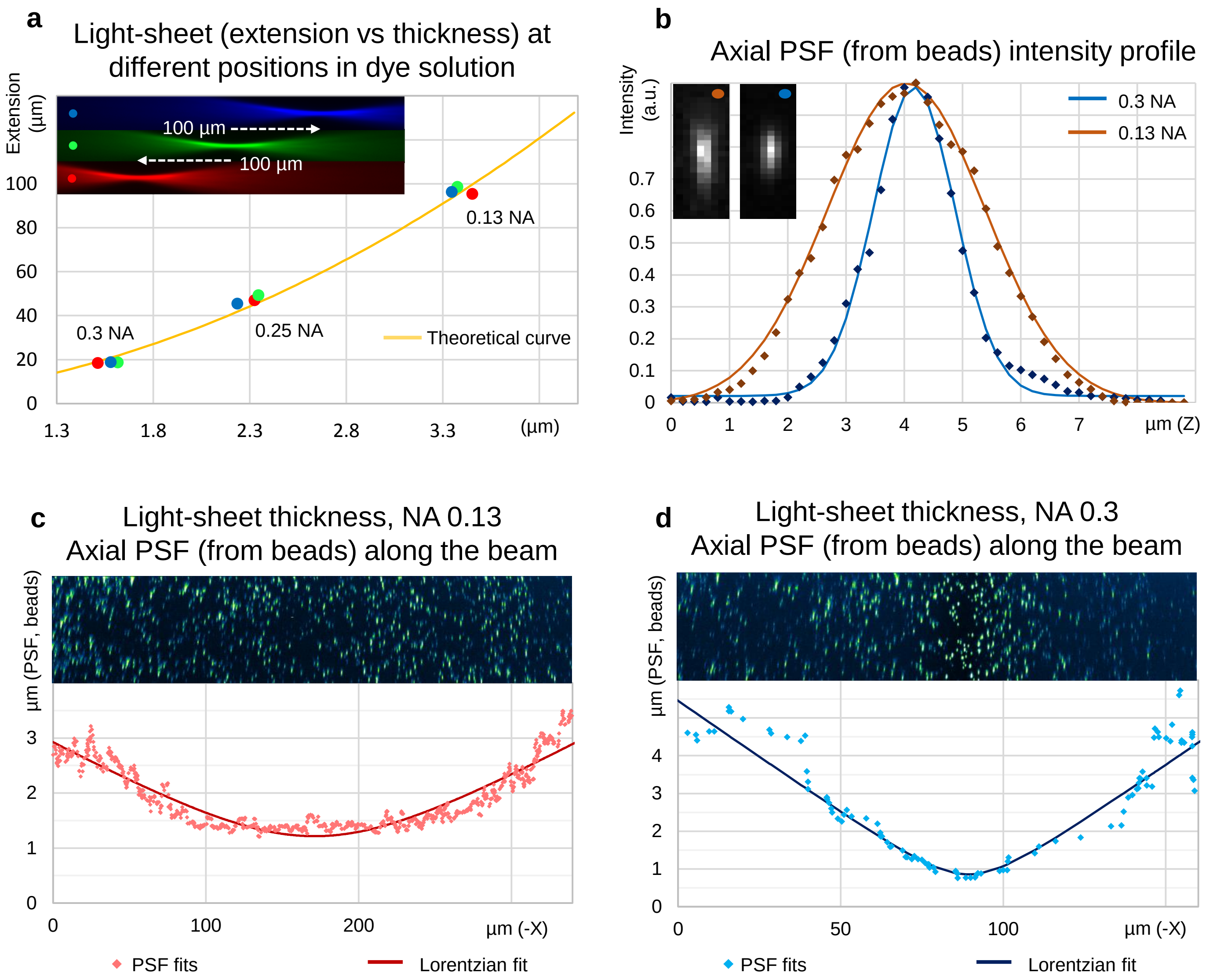

Supplementary Figure S2. Analysis of illumination beam quality. The values for light sheet thickness and extension are obtained from fitting a Gaussian and a Lorentzian, respectively, to the raw data. (a): Comparison of light sheet properties with theoretical values (for a Gaussian beam) across camera field of view. The three colours correspond to 3 different positions across the camera view, spaced 100 µm apart as visualised on the beam images (stationary beam in dye solution, see methods). Good correspondence confirms good beam quality across entire camera view. (b): Light sheet thickness profile, obtained from bead PSFs summed along detection direction. (c)(d): Illumination beam axial profiles obtained from bead PSFs (PSF extent given as Gaussian variance, σ).

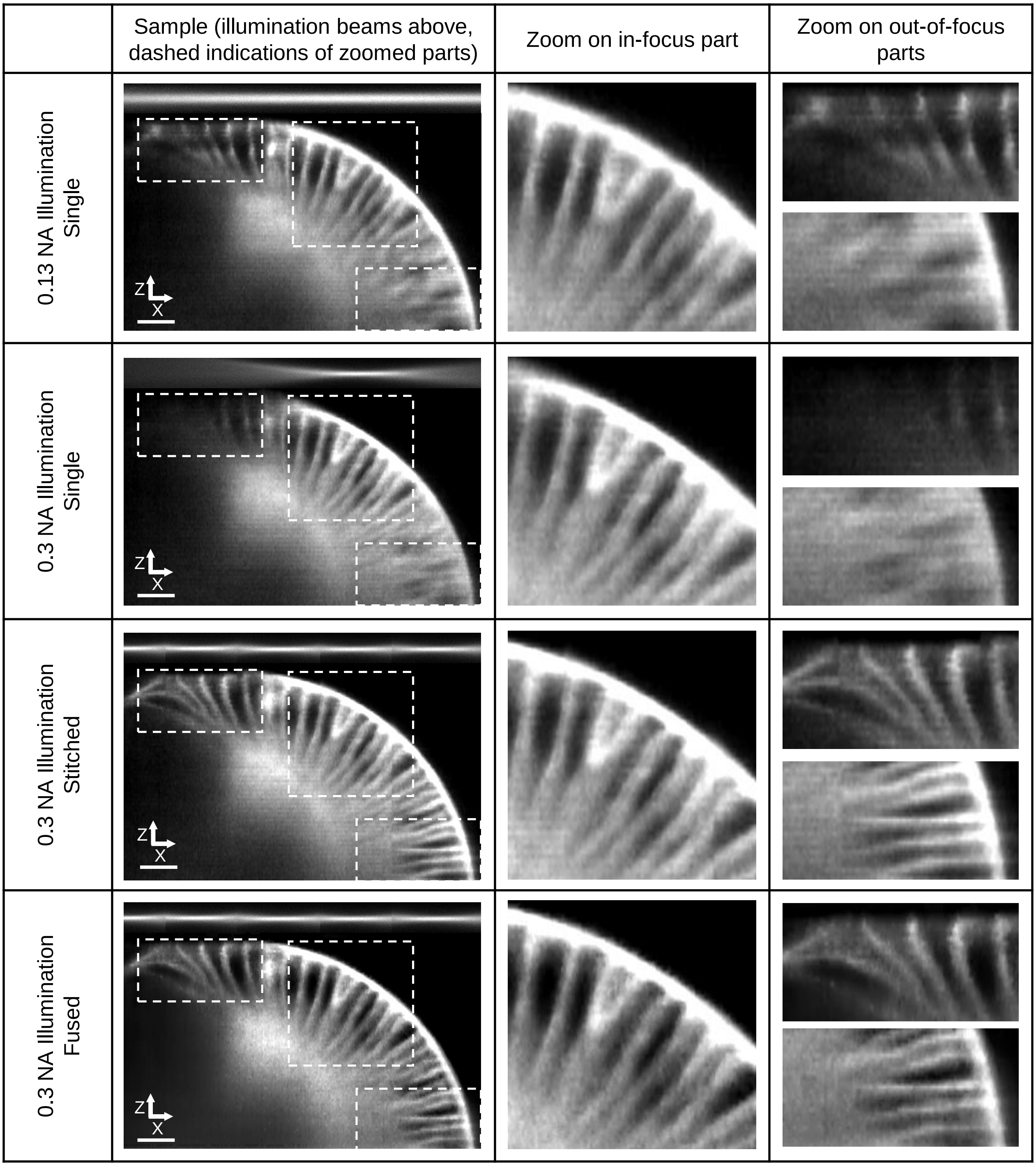

Supplementary Figure S3. Trade-off between light-sheet thickness and extension. The table shows data for Drosophila embryo, stage 6, used in Figure 3. The 0.3 NA illumination images (row 2) show improved quality over 0.13 NA illumination (row 1) around the focus of the illumination (column 2), while away from the focus this improvement is lost (column 3). When stitching (row 3) or fusing (row 4) multiple 0.3 NA images it is possible to retain high image quality across entire field of view. The images of the illumination beams with conserved relative position to the samples are shown above to better visualise the relation between light-sheet thickness, extension and image quality. All scale bars 15 µm.

Supplementary Figure S4. Acquisition orders for stitching/fusion

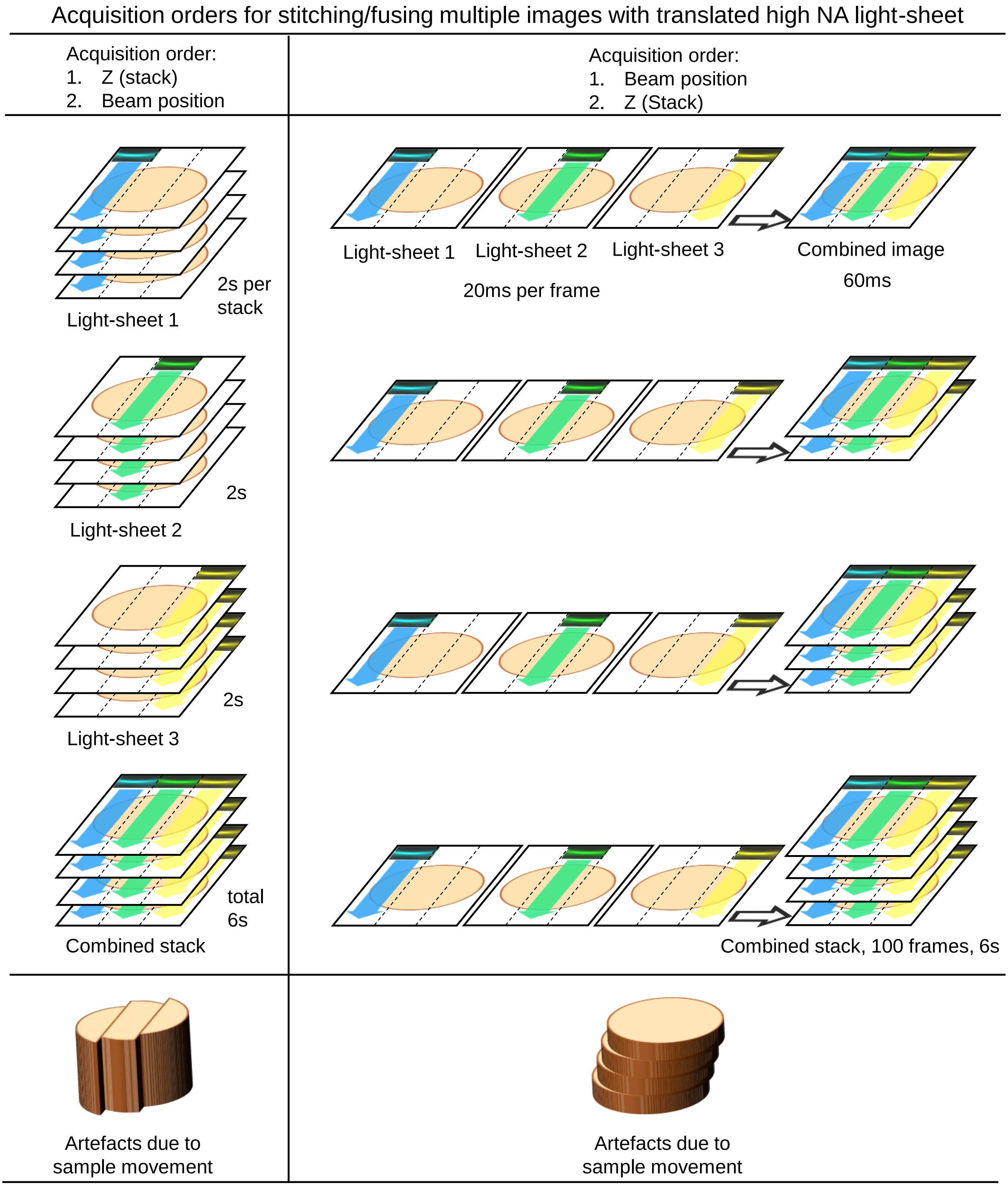

Supplementary Figure S4. Acquisition orders for stitching/fusion. When overcoming the trade-off between light-sheet extension and thickness, the conventional way is to acquire multiple stacks with translated light-sheet positions (left column). However for very fast varying samples this can create problems in combining the images, because parts of the stacks which are spatially close can have large temporal differences. The tuneable lenses, due to their speed, permit to acquire images with different light-sheet positions for each slice in the stack (right column). Hence there are no discontinuities in acquisition time making it considerably easier to stitch or fuse.

Supplementary Figure S5. Tuneable lens response

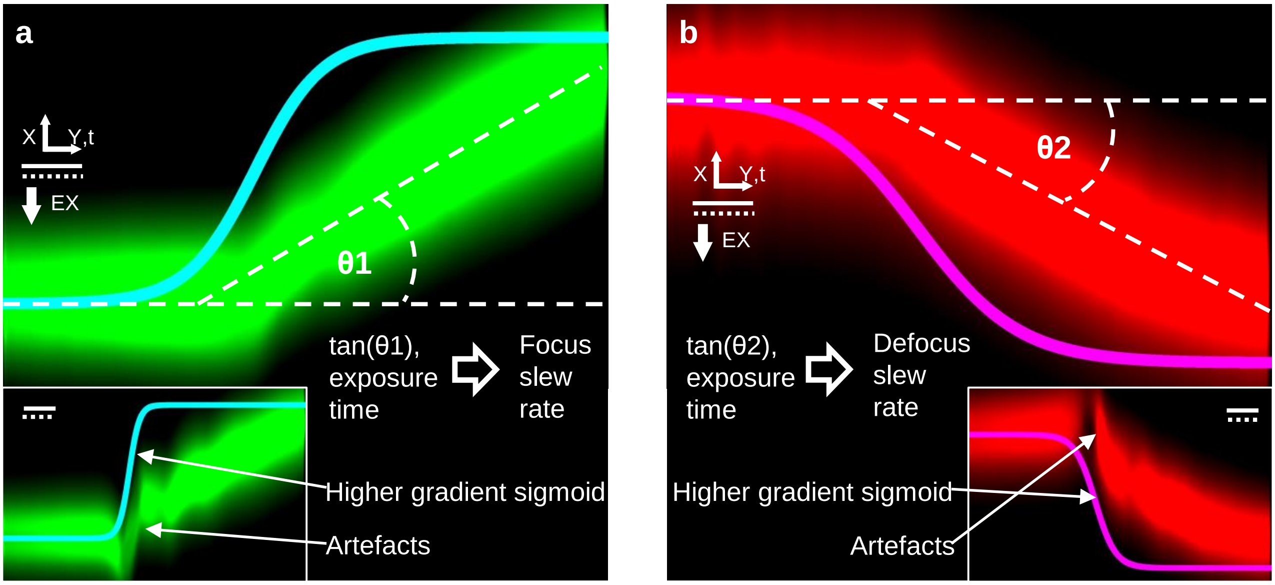

Supplementary Figure S5. Tuneable lens response. The panels visualise step response of the lenses as light-sheets (green, red), saturated at half the peak intensity (FWHM), generated using sigmoid functions. The sigmoid functions (cyan, magenta) were calibrated for the non-linear responses of the lenses (see supplementary discussion). The sigmoid functions were used as an approximation to a step function because tuneable lenses create artefacts in the light-sheets when driven with signals with very high gradients (see insets in the main panels, where steeper sigmoids were used). The Y axis (horizontal – images rotated and flipped relative to the other images in the paper) is the direction of slit propagation, whose speed was 10µs/pixel (2048 pixels, pixel - 0.264 µm). As such, the Y dimension corresponds also to time, t, while X only to space. The angles (θ1, θ2) therefore give the slew rate of the tuneable lens, which are 15.3 µm/ms for focusing (a), and 13.4 µm/ms for defocusing (b). Scale bars, solid - 50 µm, dashed – 2ms.

Supplementary Figure S6. Tuneable lens response

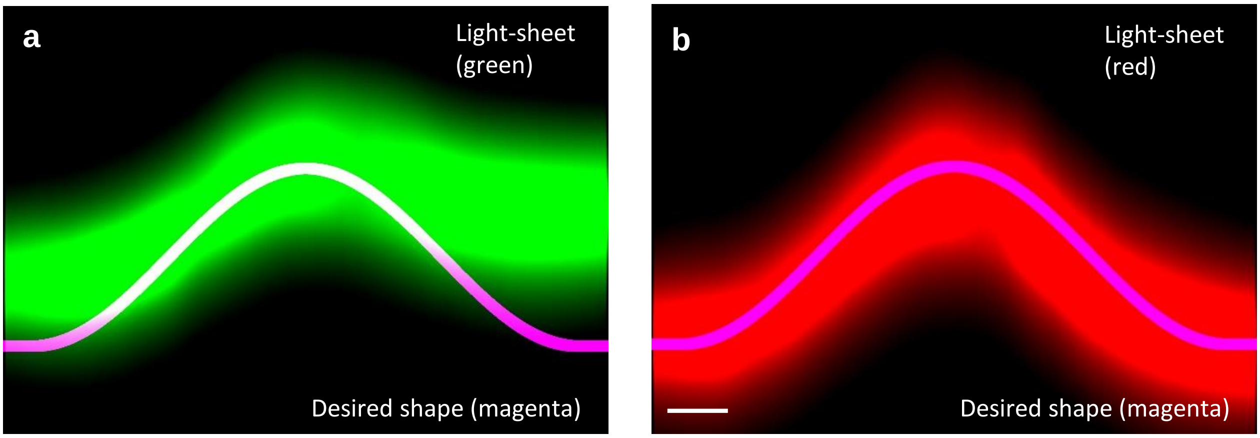

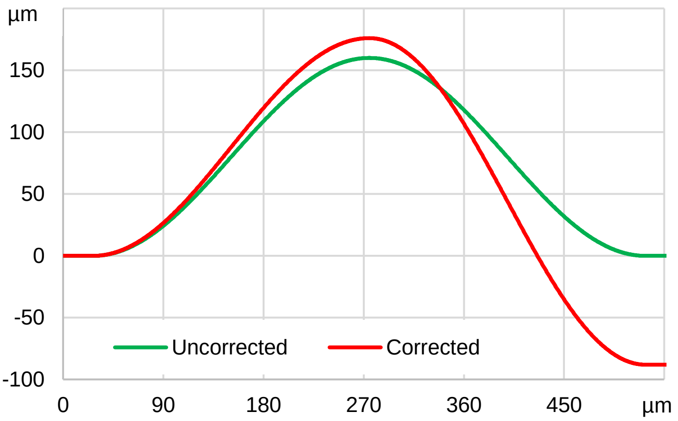

$$f_{corrected}(X) = \int_0^X f'_{desired}(x) \times p\left(f'_{desired}(x)\right) dx + f_{desired}(0)$$

$$p\left(f'_{desired}(x)\right) = \begin{cases} P_1 \quad , & f'_{desired}(x) \ge 0 \\ P_2 \quad , & f'_{desired}(x) < 0 \end{cases}$$

$$I_{lens} = f_{calibration}\left(f_{corrected}(X)\right)$$

Supplementary Figure S6. Tuneable lens response. The panels show light-sheets generated using cosine derived signals. The green light sheet is generated using a pure cosine signal (corrected only for steady state non-linear response of the lenses) indicated by magenta function. The red light sheet is generated using further corrected signal to match the desired cosine profile (magenta). The signals (after the steady state non-linear response correction) are given in the graph. The red profile (corrected) is the cosine function with enhanced positive and especially negative derivatives (see supplementary discussion) to account for unsymmetrical response of the lenses. The equations shows mathematically how the corrected signal is generated (see also Supplementary Discussion). In the desired signal ($f_{desired}$, i.e. uncorrected signal, green) positive and negative derivatives are adjusted (function p: parameter P_1 and P_2 to adjust independently the positive and negative derivatives respectively) to yield the corrected function ($f_{corrected}$, red). Then calibration data is applied to generate the current signal (I_{lens})for the lenses. All scale bars 50 µm.

Supplementary Figure S7. Light-sheet sculpting to match a sample

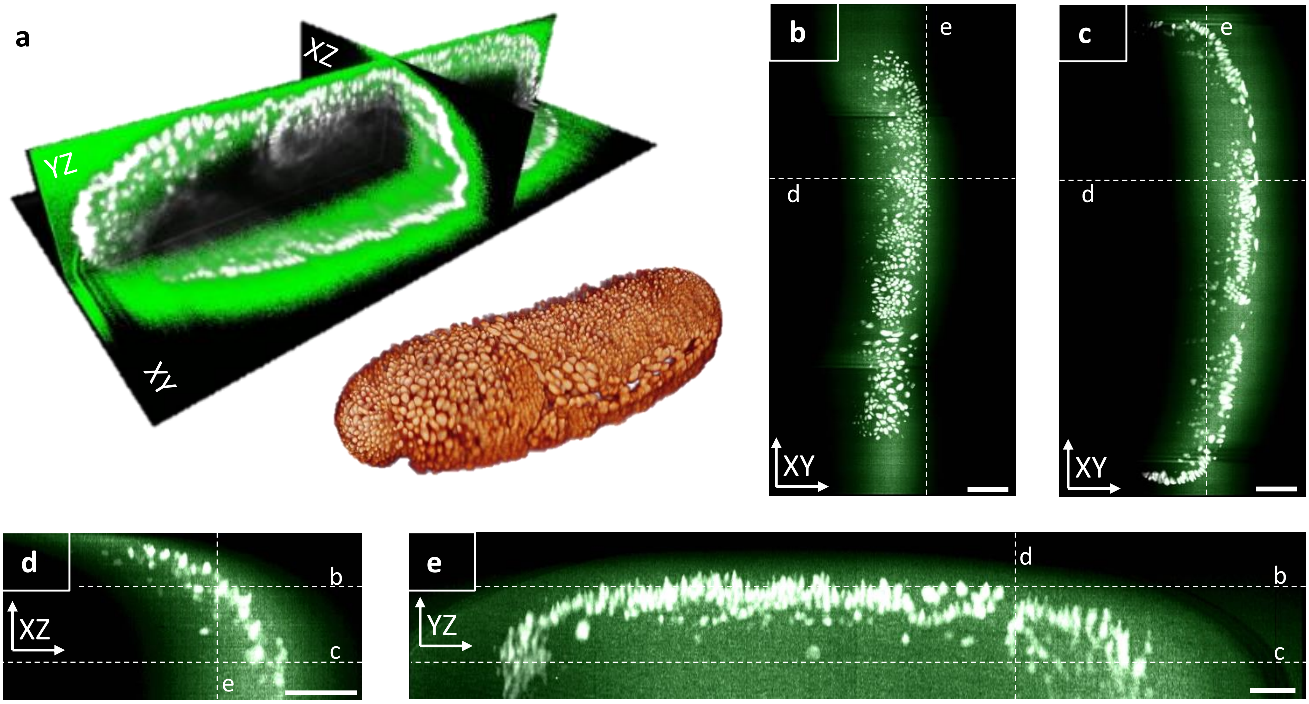

Supplementary Figure S7. Light sheet shaping to match a sample. (a): 3D rendering of nuclei labelled (histoneGFP), stage 7, *Drosophila* embryo (orange) and, above, XY, XZ and YZ cross-sections (indicated) through the sample (white) and light-sheet used to acquire the data (green). (b-e): selected cross-sections from the volume in (a) showing how the sample (white) is matched with illumination intensity (green) using tuneable lenses to shift beam focus. The dashed white lines in panels (b-e) show their relative positions. All scale bars 30µm.

Supplementary Figure S8. Eye of a 24hpf Zebrafish embryo

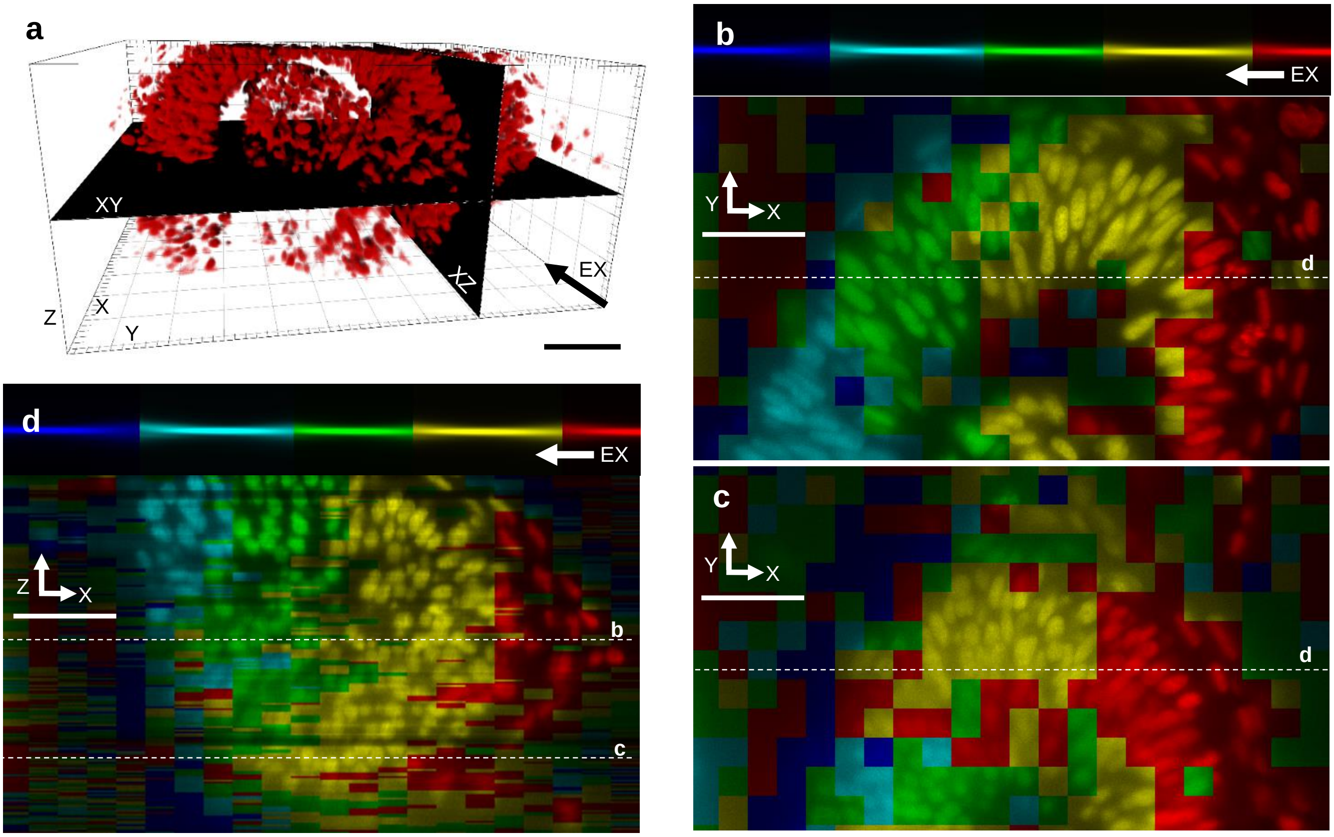

Supplementary Figure S8. Eye of a 24hpf Zebrafish embryo with GFP labelled nuclei. (a): 3D view of the eye with planes indicating cross-sections for images (b) and (d). (b)(c)(d): The contrast analysis images (see main text). These are 5 images with translated 0.3 NA beams (at the top) combined into one based on their contrast and color-coded to correspond to the beam positions. As opposed to figure 5 in the main text the combining of the images was done on a 2D panel basis as opposed to 3D boxes. This gives higher variability in colour-pattern along z direction and allows for better visualisation the effects of scattering in the detection path. Indeed, comparing the panels (b) and (c), at different depths in the stack, the colour pattern is much disrupted in the latter (deeper) one. It can be seen more linearly on the XZ cross section (panel (d), with panel (b) and (c) planes indicated). All scale bars 30µm. EX indicates excitation beam direction.

Supplementary Figure S9. Simulations of various defocusing modes

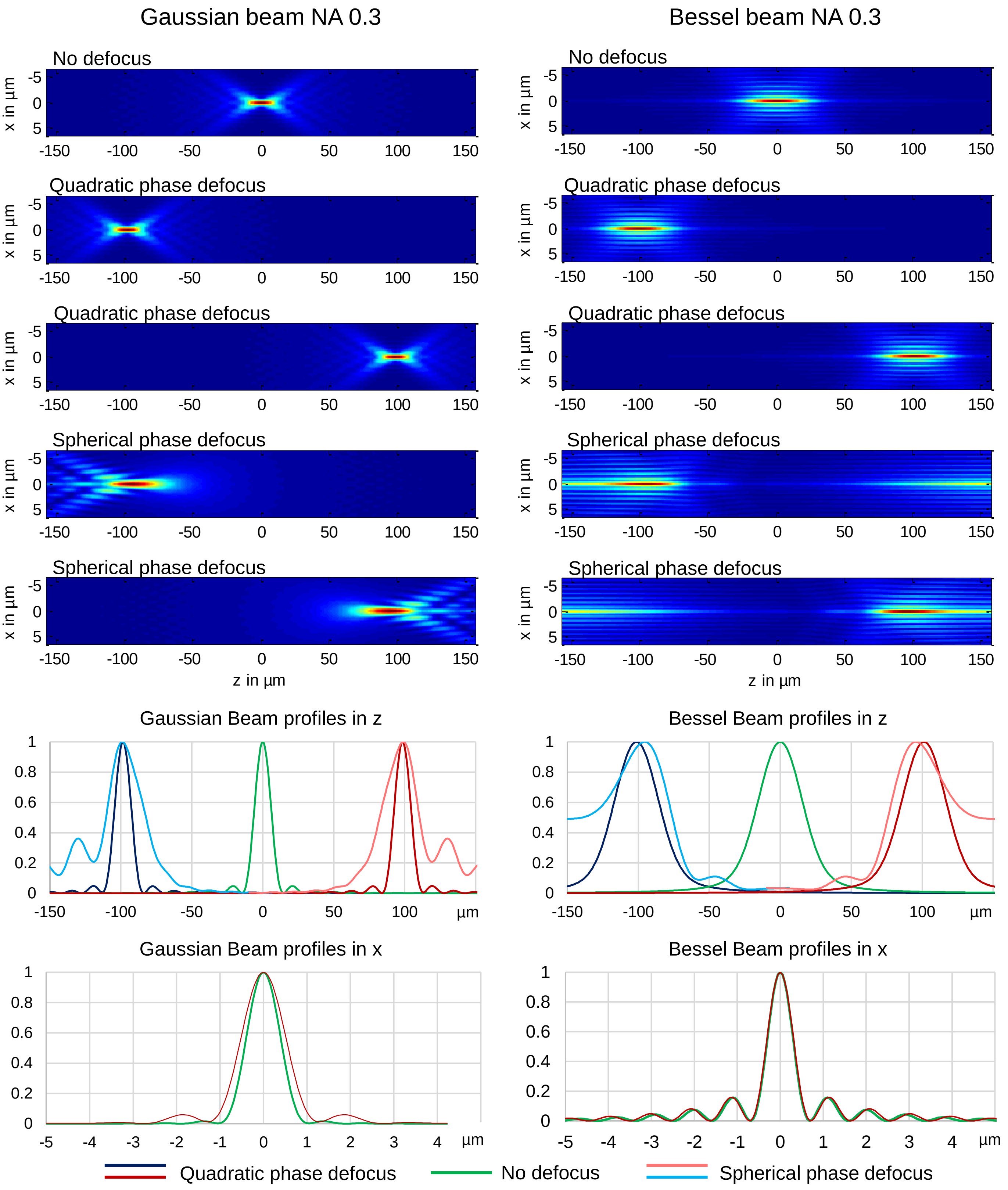

Supplementary Figure S9. Simulations of various defocusing modes. Left column shows Gaussian beam defocusing with perfect defocusing phase (square phase defocus) using, for example, an SLM and defocusing using spherical phase generated by tuneable lenses. The right column shows equivalent for Bessel beam. The graphs below show profiles through the beam figures along z and x direction. The Bessel beam suffers less artefacts than Gaussian beam when defocused using lenses (see supplementary discussion).

Supplementary Figure S10. Simulations of various defocusing

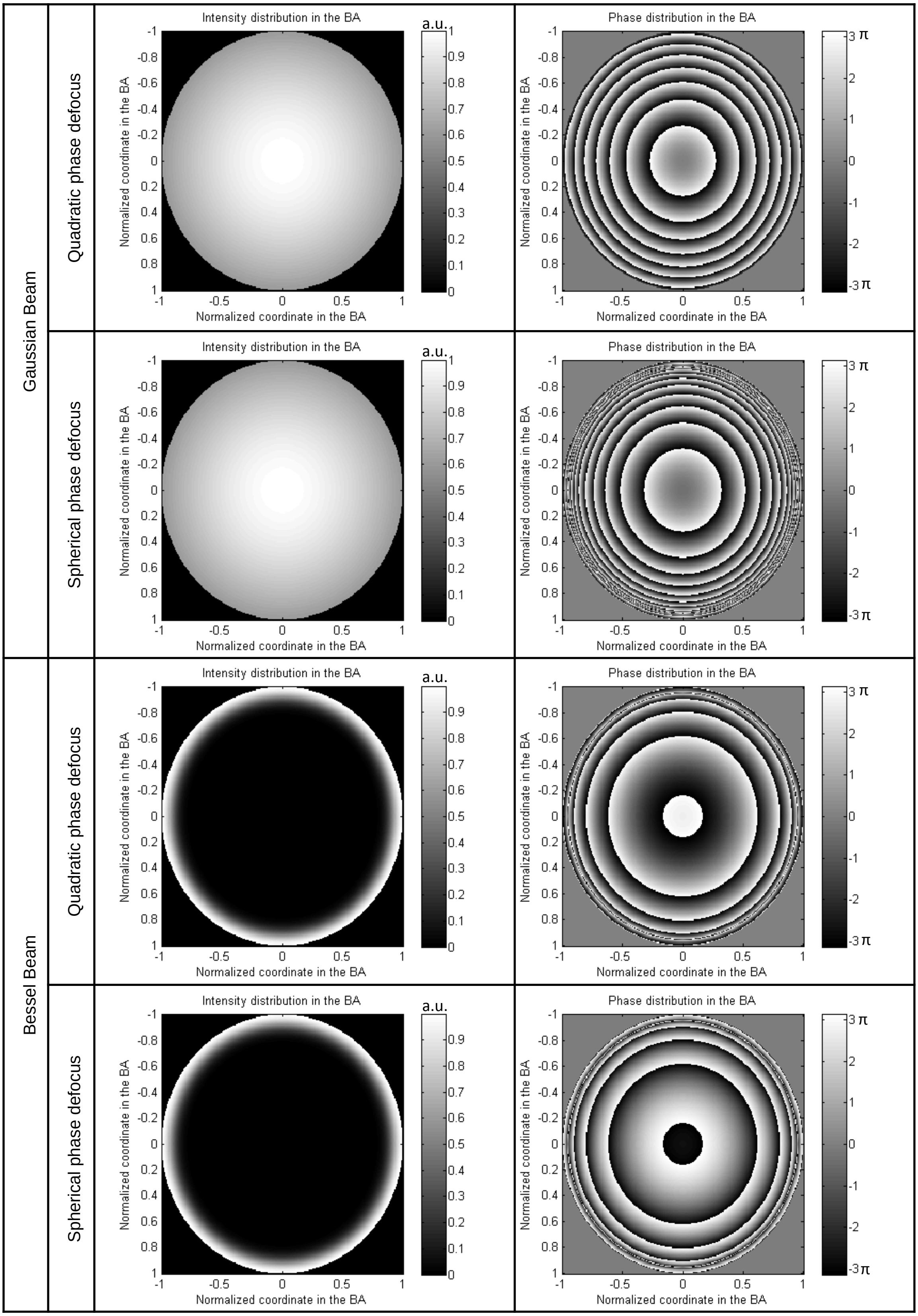

Supplementary Figure S10. Simulations of various defocusing modes (continued).The table showing intensity (left column) and phase (right column) distributions in the back aperture of the illumination objective for different beam types (Gaussian and Bessel) and defocusing modes (quadratic and spherical). The spherical phase distribution, which is only an approximation to perfect defocusing phase (quadratic) can generate artefacts (Supp. Fig. 7). For Bessel beams there is much less variation in phase in a thin intensity ring than for full circle in case of Gaussian beam hence less chance of aberrations. (see supplementary discussion).

Supplementary Movies

Supplementary Movie 1. 3D rendering of nuclei labelled (histoneGFP), stage 6, *Drosophila* embryo (as in Supplementary Figure 5) imaged using ellipsoid shaped light-sheet (Supplementary Movie 2) in slit-scanning mode. Exposure time: 20ms, illumination NA: 0.22.

Supplementary Movie 2. 3D rendering of the light sheet used to image the sample in Supplementary Movie 1.

Supplementary Movie 3. An overlay between Supplementary Movies 1 and 2.

All scale bars 30 µm
